# Supplementary material for: Gene expression profiles for in vitro human stem cell differentiation into osteoblasts and osteoclasts: a systematic review
Source: PeerJ. 2022 Oct 17;10:e14174. doi: 10.7717/peerj.14174 (PMC9583853; doi:10.7717/peerj.14174)
Supplement: Supplemental Information 2 [file peerj-10-14174-s002.docx]

**Rationale**

Studies involving the gene expression profiles on the differentiation of stem cells into osteoblast and osteoclast cells have been regularly updated on different human stem cells. However, there is a lack of knowledge on the most effective technique and genes involved to determine the differentiation of human stem cells into osteoblast and osteoclast cells. Therefore, this systematic review revolves around molecular techniques used to observe gene expression profiles on the differentiation of stem cells into osteoblast and osteoclast cells. Furthermore, genes that are commonly used are suggested to be the indication of differentiation into osteoblast and osteoclast cells respectively. Consequently, the technique and genes stated are suggested as a standard to indicate and observe the differentiation of human stem cells into osteoblast and osteoclast cells respectively.
